# Supplementary material for: Sustained microglial depletion with CSF1R inhibitor impairs parenchymal plaque development in an Alzheimer’s disease model
Source: Nat Commun. 2019 Aug 21;10:3758. doi: 10.1038/s41467-019-11674-z (PMC6704256; doi:10.1038/s41467-019-11674-z)
Supplement: Supplementary file 1 — Supplementary Information [file 41467_2019_11674_MOESM1_ESM.pdf]

Supplementary Information

Supplement to "Sustained microglial depletion with CSF1R inhibitor impairs parenchymal plaque development in an Alzheimer's disease model" by *Spangenberg et al*.

Table of Contents

Supplementary Methods ………………………………………………Page 2

Supplementary Tables (n=7) ………………………………….............Page 9

Supplementary Figures (n=10) ……………………….....................Page 16

Supplementary Methods

Synthesis of PLX5622 and formation of PLX5622-fumaric acid salts

PLX5622 was synthesized from commercially available 2-amino-6-fluoropyridine (**1**), 5-fluoro-2-methoxypyridine-3-carbaldehyde (**2**), and 3-iodo-5-methyl-1-(triisopropylsilyl)-1H-pyrrolo[2,3-b]pyridine (**7**) using the reaction scheme shown in Figure 2C.

Step 1 – Preparation of 6-fluoro-N-((5-fluoro-2-methoxypyridin-3-yl)methyl)pyridin-2-amine (3). To an appropriately sized reactor, 2-amino-6-fluoropyridine (**1**, 12 kg, 107.05 mol, 1 equiv), 5-fluoro-2-methoxypyridine-3-carbaldehyde (**2**, 16.61 kg, 107.05 mol, 1 equiv) and acetonitrile (120 L/94 kg, 10 vol.) were added. Stirring was initiated under nitrogen, and the reaction was cooled to -5 ˚C. Triethylsilane (37.34 kg, 51.29 L, 321.15 mol, 3 equiv) was added to the reaction via an addition funnel over a period of 15 minutes.

Next, trifluoroacetic acid (36.62 kg, 24.74 L, 321.15 mol, 3 equiv) was added via an addition funnel over a period of 1 hour. Note: high exotherm (~20-25 ˚C) was observed. The reaction maintained an internal temperature below 20 ˚C during the addition. Trifluoroacetic acid addition may be stopped intermittently to control the exotherm. The mixture was stirred for 10-15 minutes at ambient temperature, then heated to reflux for 6 hours. The progress of the reaction was monitored by HPLC.

The reaction mixture was cooled to room temperature, and concentrated under vacuum with a jacket temperature of 45-55 ˚C (~36 L remains in the reactor, ~1/4 volume). The reaction was diluted with water (~60 L, 5 vol.) and cooled to 0-10 ˚C; an aqueous solution of 15% ammonium hydroxide was added to adjust the pH of the solution toward basic (pH ~9), while maintaining the temperature below 20 ˚C during addition. The reaction was stirred for 2 hours at 15-20 ˚C.

The product/solid was collected onto a polypropylene filter pad. The solid was washed with water (2 × 24 L, 4 vol.) and dried on the filter for at least 15 minutes. The solid was then suspended in heptanes and stirred at ambient conditions for 5-6 hours, and filtered onto a polypropylene filter pad. The solid was washed with heptanes (2 × 12 L, 2 vol.), and then dried under high vacuum overnight at 36 ˚C to afford a yellow solid (**3**, 24.2 kg, 90%). ^{1}H-NMR (400 MHz, DMSO-d_6) δ 8.01 (d, J = 2.8 Hz, 1H), 7.52 (m, 1H), 7.42 (m, 2H), 6.42 (dd, J = 1.6, 8.0 Hz, 1H), 6.12 (dd, J = 1.6, 8.0 Hz, 1H), 4.32 (d, J = 6.0 Hz, 2H), 3.89 (s, 3H); MS (ESI) $[M+H^+]^+$ = 252.1.

Step 2 – Preparation of (6-fluoro-5-iodo(2-pyridyl))[(5-fluoro-2-methoxy(3-pyridyl))methyl]amine (4). To an appropriately sized reactor, 6-fluoro-N-((5-fluoro-2-methoxypyridin-3-yl)methyl)pyridin-2-amine (**3**, 32.5 kg, 129.36 mol, 1 equiv) in acetonitrile (227.5 L) was added and initiated stirring under nitrogen. The reaction was cooled to -5 ˚C, and N-iodosuccinimide (29.1 kg, 129.36 mol, 1 equiv) was added portion wise over a period of 30 minutes while maintaining internal temperature below 0 ˚C. The reaction was allowed to warm

to ambient conditions slowly and stir overnight (12-24 hours) at ambient conditions.

The reaction mixture was cooled to 0 ˚C and stirred for 2 hours, and the product precipitated out of the reaction. The solid was collected via filtration onto a polypropylene filter. The solid was washed with cold acetonitrile (0 ˚C, 2 × 16.25 L, 2 × 12.8 kg, 1 vol.). NOTE: the product has good solubility in acetonitrile at room temperature; thus the solvent needs to be cold to avert loss to filtrate. The solid was dried under high vacuum for 12 hours at ambient temperature to provide a light yellow solid (**4**, 23.81 kg, 61%). ^{1}H-NMR (400 MHz, DMSO-d_6) δ 8.02 (d, J = 2.8 Hz, 1H), 7.78 (m, 1H), 7.59 (m, 1H), 7.42 (dd, J = 2.4 8.8 Hz, 1H), 6.34 (d, J = 8.0 Hz, 1H), 4.30 (d, J = 5.2 Hz, 2H), 3.89 (s, 3H); MS (ESI) $[M+H^{+}]^{+}$ = 377.8.

Step 3 – Preparation of (*tert*-butoxy)-N-(6-fluoro-5-iodo(2-pyridyl))-N-[(5-fluoro-2-methoxy(3-pyridyl))methyl]carboxamide (5). To an appropriately sized reactor, (6-fluoro-5-iodo(2-pyridyl))[(5-fluoro-2-methoxy(3-pyridyl))methyl]amine (**4**, 8500 g, 22.54 mol, 1 equiv) in THF (42.5 L, 37.8 kg) were added and stirring was initiated under nitrogen. The reaction was cooled to 10-15 ˚C. Next, di-*tert*-butyl dicarbonate [$(BOC)_2O$] (5411 g, 24.79 mol, 1.1 equiv. melted in water bath) dissolved in THF (~17 L, 15.11 kg) was added over 10 minutes at ambient temperature. Dimethylaminopyridine (137.7 g, 1.13 mol, 0.05 equiv) was added portion-wise over 15 minutes maintaining reaction temperature below 20 ˚C. The reaction mixture was stirred for a minimum of 6 hours at ambient temperature.

The reaction mixture was concentrated to 1/6 of total volume, and then via co-evaporation with methyl *t*-butyl ether (MTBE); the evaporation/co-evaporation was repeated once. The reaction was diluted with MTBE and washed with a solution of ammonium chloride (12.75 kg) in water (51 L), then water (42.5 L). The reaction mixture was dried over magnesium sulfate (1.7 kg), filtered, concentrated, and then co-evaporated with dry THF (3 × 25.5 L, 3 × 22.67 kg, 3 × 3 vol.). The reaction mixture was diluted with dry THF (~85.85 L, 76.32 kg, 10.1 mL/g starting material) and used in the next step of the reaction. ^{1}H-NMR (400 MHz, DMSO-d_6) δ 8.31 (m, 1H), 8.03 (d, J = 2.8 Hz, 1H), 7.55 (d, J = 8.4 Hz, 1H), 7.29 (dd, J = 2.0, 8.4 Hz, 1H), 4.90 (s, 2H), 3.82 (s, 3H), 1.35 (s, 9H); MS (ESI) $[M+H^{+}]^{+}$ = 477.8.

Step 4 – Preparation of *tert*-butyl ((5-fluoro-2-methoxypyridin-3-yl)methyl)(6-fluoro-5-formylpyridin-2-yl)carbamate (6). To an appropriately sized cryogenic reactor, (*tert*-butoxy)-N-(6-fluoro-5-iodo(2-pyridyl))-N-[(5-fluoro-2-methoxy(3-pyridyl))methyl]carboxamide (**5**, 10.76 kg, 22.54 mol, 1 equiv) in THF (~85.85 L, 76.32 kg, telescoped from previous step already as a solution in THF) was added, and stirring was initiated under nitrogen. The reaction was cooled to -78 ˚C using a dry ice/acetone bath. Isopropylmagnesium chloride (12.96 L, 2M solution in THF, 25.92 mol, 1.15 equiv) was introduced to the reaction via an addition funnel over a period of 60 minutes, while maintaining a temperature range of -70 ˚C to -78 ˚C. The reaction stirred for an additional 2-3 hours at -70 ˚C to -78 ˚C.

DMF (4942 g, 5235 mL, 67.61 mol, 3 equiv) was added via an addition funnel over a period of

30 minutes maintaining a temperature range of -70 ˚C to -78 ˚C. The reaction was allowed to warm to 0 ˚C over 5-6 hours. A cooled solution of ammonium chloride (16.13 kg) in water (64.54 L, 64.54 kg) was introduced to the reaction mixture at 0 ˚C. The reaction was diluted with MTBE (53.78 L, 39.80 kg, 5 vol.) and mixed. The layers were separated and the aqueous layer was extracted twice with MTBE (2 × 107 L, 2 × 79.6 kg, 2 × 10 vol.). The organic layers were combined and washed with water (~32.27 L). The organic layer were then dried over $MgSO_4$ (2.15 kg). The organic layer was then filtered and concentrated to minimum stir volume after each co-evaporation. The crude material was then mixed with heptanes and stirred overnight at ambient conditions. The solid was filtered and washed with additional heptanes (1 x 10.7 L, 1 vol.). The solid was dried for 16 hours under high vacuum at 30 ˚C to provide a white solid (**6**, 5216 g, 61% over 2 steps). ^{1}H-NMR (400 MHz, DMSO-d_6) δ 10.03 (s, 1H), 8.31 (m, 1H), 8.04 (d, J = 2.8 Hz, 1H), 7.97 (d, J = 8.0 Hz, 1H), 7.31 (dd, J = 2.0, 8.8 Hz, 1H), 5.03 (s, 2H), 3.84 (s, 3H), 1.38 (s, 9H); MS (ESI) $[M+H^+]^+$ = 380.1.

Step 5 – Preparation of *tert*-butyl ((5-fluoro-2-methoxypyridin-3-yl)methyl)(6-fluoro-5-(hydroxy(5-methyl-1-(triisopropylsilyl)-1H-pyrrolo[2,3-b]pyridin-3-yl)methyl)pyridin-2-yl)carbamate (8). To an appropriately sized reactor, dry THF (~50 L, 44.46 kg, 8.7 mL/g starting material) was added and cooled at -20 ˚C to -25 ˚C. The reaction was then charged with isopropylmagnesium chloride (9850 mL, 2M solution in THF, 19.70 mol, 1.3 equiv) to the flask over a minimum of 10 minutes while maintaining the mixture at -10 ˚C to -25 ˚C. 3-iodo-5-methyl-1-(triisopropylsilyl)-1H-pyrrolo[2,3-b]pyridine (**7**, 6908 g, 16.67 mol, 1.1 equiv) dissolved in THF (~17.25 L, 15.33 kg, 3 mL/g starting material) was added to the reaction via an additional funnel over a minimum of 20 minutes while maintaining temperature of the mixture at -10 ˚C to -25 ˚C. Additional THF (2.9 L, 2.6 kg, 0.5 mL/g starting material) was used to rinse the additional funnel and added to the reaction. The reaction was allowed to warm to 0 ˚C to 5 ˚C over 0.5-1 hour to complete metal halogen exchange. Next, the reaction was cooled down to -70 ˚C to -75 ˚C with dry ice/acetone bath maintaining positive pressure of nitrogen inside the flask. A solution of *tert*-butyl ((5-fluoro-2-methoxypyridin-3-yl)methyl)(6-fluoro-5-formylpyridin-2-yl)carbamate (**6**, 5749 g, 15.15 mol, 1.0 equiv) dissolved in dry THF (~17.25 L, 15.33 kg, 3 mL/g starting material) was added to the reaction over 15 minutes, while maintaining the temperature between -65 ˚C to -75 ˚C. Additional THF was used to rinse out the addition funnel. The reaction was allowed to stir 1-5 hours, and warmed up to 0 ˚C to 5˚C.

A solution of ammonia chloride (8.62 kg, 1.5 g/g starting material) in water (34.49 L, 34.49 kg, 6 mL/g starting material) was added to the reaction mixture over a minimum of 5 minutes while maintaining temperature of the mixture at 0 ˚C to 20 ˚C. The reaction was allowed to warm to 15 ˚C to -20 ˚C. Additional stirring may be needed to ensure all solids are dissolved.

The layers were separated, and the aqueous layer was extracted with methyl *t*-butyl ether MTBE (28.75 L, 21.27 kg, 5 mL/g starting material). The organic layers were combined and washed with brine (28.75 L, 5 mL/g starting material) and dried over magnesium sulfate (1150 g, 0.2 g/g starting material) over 15-30 minutes with occasional stirring. The magnesium sulfate was

filtered off and washed with additional MTBE (2 × 2.9 L, 2 × 2.1 kg, 2 × 0.5 mL/g starting material). The reaction was then concentrated under reduced pressure to a minimum stir volume and then co-evaporated with acetonitrile (1 × 11.5 L, 1 × 9.04 kg, 1 × 2 mL/g starting material) under reduced pressure at 40 ˚C to 50 ˚C in order to remove residual THF and MTBE to arrive at a yellow oil. The residue was then mixed with acetonitrile with heating to ensure a clear (or mostly clear solution). The reaction was allowed to slowly cool down to ambient temperature with stirring over a minimum of 12 hours to ensure precipitation of the product. The slurry solution was then cooled to 0 ˚C to 5˚C for minimum of 1 hour and filtered. The collected solid was then washed with cold acetonitrile (2 × 5.75 L, 2 × 4.52 kg, 2 × 1 mL/g starting material), and dried in an vacuum oven at 35 ˚C to 40 ˚C to arrive at a constant weight to give a white to off-white solid (**8**, 8.3 kg, 82%). ^{1}H-NMR (400 MHz, DMSO-d_6) δ 8.14 (m, 1H), 8.01 (m, 2H), 7.66 (d, J = 8.0 Hz, 1H), 7.63 (s, 1H), 7.27 (dd, J = 2.4, 8.8 Hz, 1H), 7.14 (s, 1H), 6.05 (d, J = 4.4 Hz, 1H), 5.99 (d, J = 4.8 Hz, 1H), 4.88 (s, 2H), 3.74 (s, 3H), 2.29 (s, 3H), 1.75 (m, 3H), 1.35 (s, 9H), 1.00 (d, J = 7.2 Hz, 18H); MS (ESI) $[M+H^+]^+$ = 668.1.

Step 6 – Preparation of 6-fluoro-N-((5-fluoro-2-methoxypyridin-3-yl)methyl)-5-((5-methyl-1H-pyrrolo[2,3-b]pyridin-3-yl)methyl)pyridin-2-amine (PLX5622). To an appropriately sized lean reactor, triethylsilane (16154 mL, 11760 g, 2 mL/g starting material) was charged to the reactor. Trifluoroacetic acid (16154 mL, 23908 g, 2 mL/g starting material) was added in one portion to the reaction. Stirring was initiated under nitrogen. Water (4038.5 mL, 4038.5 g, 0.5mL/g starting material) was added over a minimum of 5 minutes while maintaining temperature of the mixture at 15 ˚C to 25 ˚C. *tert*-butyl ((5-fluoro-2-methoxypyridin-3-yl)methyl)(6-fluoro-5-(hydroxy(5-methyl-1-(triisopropylsilyl)-1H-pyrrolo[2,3-b]pyridin-3-yl)methyl)pyridin-2-yl)carbamate (**8**, 8077 g, 12.11 mol) was introduced to the reaction slowly in portions over a minimum of 30 minutes while maintaining the reaction mixture at 15 ˚C to 25 ˚C. The reaction was stirred at 15 ˚C to 25 ˚C over a minimum of 24 hours under gentle nitrogen.

The reaction was cooled to 10 ˚C to 15 ˚C. Next, ammonia solution, prepared from conc. aq. NH_3 (16154 mL, 14539 g, 2 mL/g starting material), water (32308 mL, 4 mL/g starting material) and ethanol (48462 mL, 38237 g, 6 mL/g starting material) was added to the reaction over a minimum of 30 minutes, while maintaining temperature of the mixture at 10 ˚C to 20 ˚C. The reaction was then warmed to 50 ˚C to 60 ˚C under nitrogen. Heating was then stopped and the reaction was allowed to cool to ambient conditions over 4 hours. The product was then collected via filtration. The filtered solid was then washed with ethanol (2 × 4039 mL, 2 × 3186 g, 2 × 0.5 mL/g starting material) and DI-water (2 × 8077 mL, 2 × 1 mL/g starting material) sequentially. The filtered solid was then transferred to a flask and mixed with DI-water (32308 mL, 4 mL/g starting material). The slurry was then stirred at ambient temperature for a minimum of 1 hour. The solid was collected via filtration and rinsed with DI-water (2 × 8077 mL, 2 × 1 mL/g starting material) and ethanol (2 × 4039 mL, 2 × 3184 g, 2 × 0.5 mL/g starting material). The solid was mixed with ethanol (32308 mL, 25491 g, 4 mL/g starting material), and subject to heating 65 ˚C to 75 ˚C under gentle nitrogen flow. The slurry was allowed to cool to ambient conditions over

12 hours. The solid was filtered and rinsed with ethanol (2 × 4038.5 mL, 2 × 3186.4 g, 2 × 0.5 mL/g starting material). The product was dried in a vacuum oven to afford a white to off-white solid (**PLX5622**, 3.5 kg, 73%). m.p.: 189 °C; ^{1}H-NMR (400 MHz, DMSO-d_6) δ 11.21 (s, 1H), 8.00 (s, 2H), 7.59 (s, 1H), 7.39 (m, 2H), 7.19 (m, 1H), 7.16 (m, 1H), 6.37 (d, J = 8.4 Hz, 1H), 4.29 (d, J = 6.0 Hz, 2H), 3.88 (s, 3H), 3.78 (s, 2H), 2.31 (s, 3H); ^{13}C-NMR (100 MHz, DMSO-d_6) δ 161.7, 159.4, 157.7, 156.7 (m), 154.4, 147.8, 143.8, 142.6 (d, J = 6.1 Hz), 130.9 (d, J = 25.7 Hz), 126.6, 124.6, 124.3 (m), 123.7, 119.2, 111.5, 107.4 (d, J = 30.4 Hz), 105.5, 54.1, 23.3, 18.6; MS (ESI) $[M+H^+]^+$ = 396.0.

Step 7 – Preparation of <u>PLX5622-fumaric acid salt</u> (<u>PLX5622-FA</u>). Into a 12L round bottom flask equipped with a mechanical stirrer, thermocouple, nitrogen inlet, reflux condenser and guard tube, 6-fluoro-N-((5-fluoro-2-methoxypyridin-3-yl)methyl)-5-((5-methyl-1H-pyrrolo[2,3-b]pyridin-3-yl)methyl)pyridin-2-amine **(PLX5622**, 200 g, 0.506 mol, 1 equiv) and fumaric acid (29.35 g, 0.253 mol, 0.50 equiv) in methyl ethyl ketone (3000 mL, 15 vol.) were combined and stirred under gentle nitrogen flow. The reaction was heated to reflux (75 ˚C to 83˚C). The heating was held at reflux for 10 minutes.

The hot solution was polish filtered through in line filter into another pre-heated flask/reactor (50 ˚C to 60 ˚C). The solution was heated at 70 ˚C to 75 ˚C to dissolve any solids that precipitated during the filtration. Once a clear solution is obtained, the heating stopped and the reaction was allowed to cool slowly (~10 ˚C/1 hour) to 60 ˚C to 62 ˚C. The solution was seeded with seed crystals (~1 g, 0.5 equiv by weight) suspended in methyl ethyl ketone (MEK, ~5 mL, 5 volume to weight of seed crystals) at 58 ˚C to 60 ˚C.

The solution was then cooled at a rate of 10 ˚C/1 hour, with stirring, until 0 ˚C to -5 ˚C. The solid was collected via filtration. The solid was washed with MEK (1 x 400 mL, 2 vol.). The solid was dried on filter for 1 hour, then high vacuum at 30 ˚C until constant weight was achieved. m.p.: 184 °C; ^{1}H-NMR (400 MHz, DMSO-d_6) δ 13.12 (br, 1H), 11.21 (s, 1H), 8.00 (s, 2H), 7.59 (s, 1H), 7.38 (m, 2H), 7.19 (m, 1H), 7.15 (s, 1H), 6.61 (s, 1H), 6.36 (d, J = 8.0 Hz, 1 H), 4.29 (d, J = 6.0 Hz, 2H), 3.88 (s, 3H), 3.78 (s, 2H), 2.31 (s, 3H); ^{13}C-NMR (100 MHz, DMSO-d_6) δ 166.4, 161.7, 159.4, 157.7, 156.7 (m), 154.4, 147.8, 143.7, 142.6 (d, J = 6.1 Hz), 134.4, 130.9 (d, J = 25.7 Hz), 126.6, 124.7, 124.3 (m), 123.7, 119.2, 111.5, 107.4 (d, J = 30.4 Hz), 105.5, 54.1, 23.3, 18.6; MS (ESI) $[M+H^+]^+$ = 396.1.

Formulation and dosing instructions

<u>Preparation of gavage dosing suspensions for PLX5622-FA</u>

PLX5622-FA was dissolved in DMSO at a concentration that was 20x the final dosing solution. This compound stock was stored at room temperature, protected from light. A fresh stock was made each week.

The components of the diluent generally were prepared a day or more in advance because they

took time to dissolve completely: a) 2% hydroxypropyl methyl cellulose (HPMC): 2.0 g powder brought to 100 mL deionized water; b) 25% Polysorbate 80 (PS80): 25 g brought to 100 mL deionized water. To make 100 mL diluent, add 25 mL of 2% HPMC stock (0.5% final) and 4 mL of 25% PS80 stock (1% final) to 71 mL deionized water to have final 100 mL. Final composition after mixing with compound: 0.5% HPMC, 1% PS80, 5% DMSO.

On each dosing day, the compound stock was diluted 20-fold as follows: 19 volumes of diluent were measured into the tube, and 1 volume of the 20x compound/DMSO stock was added. The cap was closed and the content of the tube was mixed by inversion and placed in a sonicating water bath to make a uniform suspension.

Preparation of PLX5622-FA dry blend

The PLX5622-FA formulated blend (dubbed PLX5622-FA-CC) is shown in Table 1.

PLX5622-FA was sieved through a 425 µm mesh sieve. In the meantime, Poloxamer 407 and Crospovidone XL10 are weighed and dispense into an appropriately-sized mortar. Mix until uniformly blended. The sieved PLX5622-FA was introduced in small portions to the blend of Poloxamer 407 and Crospovidone XL10 at a rate of 2-3 min per addition. Use the pestle and apply gentle pressure to the mixture in a circular motion for approximately 5 minutes. Mix the formulated blend another 2-3 minutes to break up potential agglomerates. Magnesium sterate was added and mix well until uniformly blended.

Chow manufacturing

PLX5622 drug chow was prepared by a trained diet preparation operator at Research Diets, Inc (New Brunswick, NJ). Each ingredient was carefully weighed to within ±0.5% of the amount specified. First PLX5622-FC-CC was thoroughly mixed with a premix and dye (the dye assured that the compound was uniformly mixed in the subsequent blend). All ingredients were then homogeneously blended according to the company's proprietary procedures. The diet blend was then pelleted; pellet quality and various other parameters were continually monitored throughout the process. Upon completion of the pelleting stage, the diet was placed into a temperature and humidity controlled room to remove moisture. After two days drying, the diet was inspected and removed from drying room for sampling and packaging. When all inspections were completed, the diet was double-packaged in plastic bags with gooseneck knots and sealed in the corrugated cardboard box. An industry recognized standard of a six-month expiration date from date of manufacture was assigned by the manufacturer.

In vitro characterization

CSF1R co-crystallization with PLX5622 was performed as previously described (Zhang et al. PNAS April 2, 2013 110 (14) 5689-5694; https://doi.org/10.1073/pnas.1219457110). X-ray diffraction data were collected at beamline 8.3.1 at the Advanced Light Source (Lawrence

Berkeley Laboratory). Crystallographic data and refinement statistics are given in Supplementary Table 2.

The in vitro kinase activities were determined by measuring phosphorylation of a biotinylated substrate peptide. Kinome selectivity screen was carried out using commercial kinase profiling service. PLX5622 was tested against a panel of 200 kinases at concentrations of 1 μM in duplicate. Kinases inhibited by over 50% were followed up by IC_{50} determination (Supplementary Table 3). Cell-based assays to evaluate the ability of PLX5622 to inhibit CSF1R, KIT and FLT3 catalytic activity in cells were conducted as previously described (Zhang et al. PNAS April 2, 2013 110 (14) 5689-5694; https://doi.org/10.1073/pnas.1219457110) (Supplementary Table 4).

Pharmacokinetics

To determine the pharmacokinetics of PLX5622 in preclinical species (mouse, rat, dog and monkey), PLX5622 was administered as a single dose intravenously and at 45 mg/kg by oral gavage to groups of 6 animals (3 per gender). These studies revealed low systemic clearance, moderate volume of distribution, and favorable oral bioavailability (F > 30%) in all four species (Supplementary Table 5).

PK of PLX5622 administered as rodent chow was evaluated in mice (Supplementary Table 6). Mice were fed with two doses (300 ppm and 1200 ppm) of PLX5622 formulated into rodent diet. Following repeated dosing, systemic exposures in mice increased in a dose-dependent manner with area under the concentration-time curve (AUC_{0-24}) exceeding 200,000 ng•hr/mL at the highest dose (Supplementary Table 6).

PLX5622 concentrations in plasma and cerebellum were analyzed for pharmacokinetic (PK) data by Integrated Analytical Solutions, Inc. (Berkeley, CA, USA).

Supplementary Tables

Supplementary Table 1. Composition of PLX5622-FA dry blend

Component:	% w/w
PLX5622-FA	78.00
Poloxamer 407	20.00
Crospovidone XL10	1.50
Magnesium Stearate	0.50
Total	100.00

Supplementary Table 2. Kinase selectivity profile [a]

Kinase	CSF1R	FLT3	KIT	AURKC	KDR
IC_{50} (μM)	0.016	0.39 [b]	0.86	1.0	1.1

Kinases with IC_{50} >1μM

ABL1, ABL2_(Arg), ACVR1B_(ALK4), ADRBK1_(GRK2), ADRBK2_(GRK3), AKT1_(PKBa), AKT2_(PKBb), AKT3_(PKBg), ALK, AMPK_A1/B1/G1, AMPK_A2/B1/G1, AURKA_(Aurora_A), AURKB_(Aurora_B), AURKC_(Aurora_C), AXL, BLK, BMX, BRSK1_(SAD1), BTK, CAMK1, CAMK1D_(CaMKI_delta), CAMK2A_(CaMKII_alpha), CAMK2B_(CaMKII_beta), CAMK2D_(CaMKII_delta), CAMK4_(CaMKIV), CDC42_BPA_(MRCKA), CDC42_BPB_(MRCKB), CDK1/CyclinB, CDK2/CyclinA, CDK5_p25, CDK5_p35, CDK7/CyclinH/MNAT1, CDK9/CyclinT1, CHEK1_(CHK1), CHUK_(IKKa), CLK2, CSF1R_(FMS), CSK, CSNK1A1_(CK1_alpha_1), CSNK1D_(CK1_delta), CSNK1E_(CK1_epsilon), CSNK1G1_(CK1_gamma_1), CSNK1G2_(CK1_gamma_2), CSNK1G3_(CK1_gamma_3), CSNK2A1_(CK2_alpha_1), CSNK2A2_(CK2_alpha_2), DAPK1, DAPK3_(ZIPK), DYRK1A, DYRK1B, DYRK3, DYRK4, EEF2K, EGFR_(ErbB1), EPHA1, EPHA2, EPHA3, EPHA5, EPHA8, EPHB1, EPHB2, EPHB3, EPHB4, ERBB2_(HER2), ERBB4_(HER4), FER, FES_(FPS), FGFR1, FGFR2, FGFR3, FGFR4, FGR, FLT3, FLT4_(VEGFR3), FRAP1_(mTOR), FRK_(PTK5), FYN, GRK4, GRK5, GRK6, GRK7, GSK3A, GSK3B, HCK, HIPK1_(Myak), HIPK2, HIPK4, IGF1R, IKBKB_(IKK_beta), IKBKE_(IKK_epsilon), INSR, IRAK4, ITK, JAK2, JAK3, KDR_(VEGFR2), LCK, LRRK2, LTK_(TYK1), LYN_A, LYN_B, MAP4K2_(GCK), MAP4K4_(HGK), MAP4K5_(KHS1), MAPK1_(ERK2), MAPK12_(p38_gamma), MAPK13_(p38_delta), MAPK3_(ERK1), MAPKAPK2, MAPKAPK3, MAPKAPK5_(PRAK), MARK1_(MARK), MARK2, MARK3, MARK4, MATK_(HYL), MELK, MERTK_(cMER), MET, MINK1, MST1R_(RON), NEK1, NEK2, NEK6, NEK9, NTRK2_(TRKB), NTRK3_(TRKC), PAK2_(PAK65), PAK3, PAK4, PAK6, PAK7_(KIAA1264), PDGFRA_(PDGFR_alpha), PDGFRB_(PDGFR_beta), PHKG1, PHKG2, PI4KB_(PI4K_beta), PIK3C3_(hVPS34), PIK3CA/PIK3R1_(p110a/p85a), PIK3CD/PIK3R1_(p110d/p85a), PIK3CG_(p110g), PIM1, PIM2, PKN1_(PRK1), PLK1, PLK2, PLK3, PRKACA_(PKA), PRKCA_(PKC_alpha), PRKCB1_(PKC_beta_I), PRKCB2_(PKC_beta_II), PRKCD_(PKC_delta), PRKCE_(PKC_epsilon), PRKCG_(PKC_gamma), PRKCH_(PKC_eta), PRKCI_(PKC_iota), PRKCN_(PKD3), PRKCQ_(PKC_theta), PRKCZ_(PKC_zeta), PRKD1_(PKC_mu), PRKD2_(PKD2), PRKG1, PRKG2_(PKG2), PRKX, PTK2B_(PYK2), RET, RET_V804L, RET_Y791F, ROCK1, ROCK2, RPS6KA1_(RSK1), RPS6KA2_(RSK3), RPS6KA3_(RSK2), RPS6KA4_(MSK2), RPS6KA5_(MSK1), RPS6KA6_(RSK4), RPS6KB1_(p70S6K), SGK_(SGK1), SGK2, SGKL_(SGK3), SNF1LK2, SPHK1, SRC, SRC_N1, SRPK1, SRPK2, STK22B_(TSSK2), STK22D_(TSSK1), STK25_(YSK1), STK4_(MST1), SYK, TBK1, TEK_(Tie2), TYRO3_(RSE), YES1, ZAP70.

[a] The inhibition screen of 200 kinases was carried out under contract at Invitrogen Life Technologies (Madison, WI) as part of their SelectScreen profiling service.

[b] FLT3 inhibition was not observed in cellular context (Supplementary Table 3)

Supplementary Table 3. Biochemical and cellular activities[a] of PLX3397 and PLX5622 (μM)

Assays	PLX3397	PLX5622	Description
CSF1R-related			
CSF1R (biochemical)	0.013 (0.012 – 0.014)	0.016 (0.012 – 0.021)	Enzymatic assay with 100μM ATP
Ba/F3 BCR-CSF1R	0.0085 (0.0077 – 0.0094)	0.017 (0.015 – 0.021)	BCR-CSF1R fusion dependent proliferation of Ba/F3 cells
Ba/F3 BCR-CSF1R (+ IL3)	>10	>10	IL3 dependent proliferation of BCR-CSF1R fusion Ba/F3 cells (control)
M-NFS-60 (+CSF1)	0.28 (0.24 – 0.33)	0.26 (0.21 – 0.32)	CSF1-CSF1R dependent proliferation of murine leukemic cell line M-NFS-60
EOC20 (+CSF1)	0.015 (0.0089 – 0.026)	0.11 (0.063 – 0.21)	CSF1-CSF1R dependent proliferation of murine microglial cell line EOC20
THP-1 CSF1R phosphor-Tyr	0.032 (0.021 – 0.050)	0.058 (0.032 – 0.11)	CSF1R autophosphorylation in human acute monocytic leukemia cell line THP-1
KIT-related			
KIT (biochemical)	0.027 (0.025 – 0.029)	0.86 (0.59 – 1.3)	Enzymatic assay with 100μM ATP
Ba/F3 BCR-KIT	0.11 (0.094 – 0.13)	2.7 (2.1 – 3.5)	BCR-KIT fusion dependent proliferation of Ba/F3 cells
Ba/F3 BCR-KIT (+IL3)	>10	>10	IL3 dependent proliferation of BCR-KIT fusion Ba/F3 cells (control)
M-07e (+SCF)	0.10 (0.086 – 0.12)	4.6 (3.5 – 6.1)	SCF-KIT dependent proliferation of human acute leukemic cell line M-07e
FLT3-related			
FLT3 (biochemical)	0.012 (0.010 – 0.014)	0.39 (0.22 – 0.69)	Enzymatic assay with 100μM ATP
Ba/F3 FLT3-ITD	0.13 (0.11 – 0.15)	>10	FLT3-ITD dependent proliferation of Ba/F3 cells
Ba/F3 FLT3 (+FLT3LG)	1.6 (1.5 – 1.8)	>10	FLT3 ligand-FLT3 dependent proliferation of Ba/F3 cells

[a] IC_{50}s are presented as geometric means and Student's t-based 95% confidence intervals (CIs; two-sided). Each IC_{50} listed was a geometric mean of $n \geq 6$ independent measurements. The 95% confidence interval for the IC_{50} was obtained by first calculating the 95% confidence interval for the logarithm of n measured IC_{50}s using the t distribution with (n-1) degrees of freedom and then back transforming using antilog. PLX5622 shows similar potency as PLX3397 in CSF1R-dependent assays. In contrast, PLX5622 is ~30 fold less potent than PLX3397 in KIT-dependent assays.

Supplementary Table 4. Crystallographic data and refinement statistics

	**CSF1R-PLX5622 (PDB: 6N33)**
**Data Collection**	
Resolution range (Å)	59.3-2.25
Spacegroup	$P4_32_12$
Unit cell (Å)	a=b=62.6, c=182.4
Number of observations	150,535
Number of unique reflections	18,091
Completeness (%)[a]	99.9 (99.9)
R_{sym}(%)[a and b]	10.0 (65.0)
Redundancy	8.3
**Refinement statistics**	
Resolution range (Å)	59.3-2.25
Number of reflections: working/free	17,092/919
R factor (%)[c]: working/free	18.1/22.0
RMSD[d] from ideality	
Bond length (Å)	0.009
Bond angles (°)	0.955
Most favored region (%)[e]	96.5
Additional allowed region (%)[e]	3.1
Disallowed region (%)[e]	0.35

a Numbers in parentheses represent values in the highest resolution shell.

b $R_{sym}=\frac{\sum_h \sum_n |I - \langle I\rangle|}{\sum_h \sum_n \langle I\rangle}$, where *I* is observed integrated intensity and <*I*> is the averaged integrated intensity taken over *n* measurements for reflection *h*.

c R factor$=\frac{\sum_h ||Fo|-|Fc||}{\sum_h |Fo|}$, where F_o is the observed structure factor amplitude and F_c is the calculated structure factor amplitudes based on the refined atomic positions, taken over the *h* reflections in the observed data set.

d Root mean square deviation.

e In the Ramachandran plot.

Supplementary Table 5. Summary of single dose pharmacokinetic parameters of PLX5622 in mouse, rat, dog, and monkey

Species	IV					PO (gavage)			
	Dose (mg/kg)	$AUC_{0-\infty}$ (ng•hr/mL)	CL (mL/min/kg)	Vss (L/kg)	$t_{1/2}$ (hr)	Dose (mg/kg)	$AUC_{0-\infty}$ (ng•hr/mL)	Cmax (ng/mL)	F
Mouse	1.92	15,500	2.1	0.34	2.6	45	215,000	26,300	59%
Rat (male)	1.13	2,630	7.7	1.2	2.3	45	99,600	12,000	95%
Rat (female)	1.13	5,110	3.7	1.0	3.9	45	181,000	15,600	89%
Dog	1.00	6,230	3.0	2.3	15	45	96,500	3,630	34%
Monkey	1.35	2,100	11	1.6	2.2	ND	ND	ND	ND

ND, not determined.

Supplementary Table 6. Steady state PK parameters of PLX5622 administered in rodent chow

Drug Chow	Mean Plasma Concentration (ng/mL)	Mean Brain Concentration (ng/mL)	Estimated $AUC_{0\text{-}24}$ in Plasma (ng•hr/mL)	Estimated $AUC_{0\text{-}24}$ in Brain (ng•hr/mL)	Brain to Plasma Ratio
300 ppm (N=6) [a]	4,340 (3,430 – 5,490)	963 (675 – 1,370)	104,000	23,100	0.22 (0.16 – 0.31)
1200 ppm (N=8)	9,090 (7,280 – 11,400)	2,390 (1,840 – 3,100)	218,000	57,300	0.26 (0.21 – 0.32)

[a] PK data using samples collected in Feng et al. J Neuroinflammation 2016 (https://doi.org/10.1186/s12974-016-0671-y). Six mice were freely fed with PLX5622-300 ppm chow for 14 days. Their plasma or brain samples were collected and analyzed for PK.

[b] PK data from this study. Paired (plasma and brain) PK samples were available for eight 5xFAD mice who were freely fed PLX5622-1200 ppm chow for 180 days.

Supplementary Table 7. Physicochemical properties of PLX3397 and PLX5622

Physicochemical properties	PLX3397	PLX5622
Molecular weight (Da)	417.82	395.41
pKa	5.2	4.14
LogD (octanol to water partition coefficient)	3.35	3.92
Caco-2 permeability (cm/s)	1.4×10^{-6}	12.2×10^{-6}

Supplementary Figures:

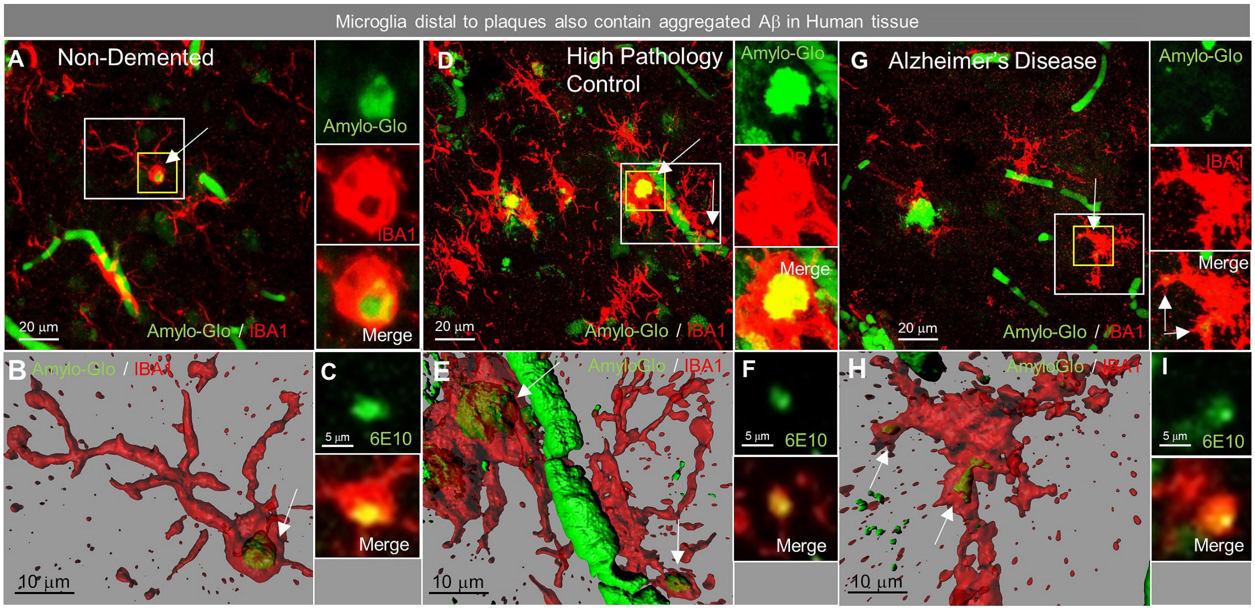

Supplementary Fig. 1: Plaque-distal microglia contain aggregated Aβ in the human brain. ***A, D, G,*** Representative images from human cortical sections of non-demented, high pathology non-demented, and AD subjects, respectively, stained for Aβ plaques (Amylo-Glo in green) and microglia (IBA1 in red). ***B, E, H,*** Three-dimensional reconstruction of microglia (IBA1 in red) and aggregated Aβ (Amylo-Glo in green), showing plaque-distal microglia containing Aβ. ***C, F, I,*** Representative images from human cortical sections of non-demented, high pathology non-demented, and AD subjects, respectively, stained for Aβ plaques (6E10 in green) and microglia (IBA1 in red). Scale bars: (A,D,G) 20 µm; (B,E,H) 10 µm; (C,F,I) 5 µm.

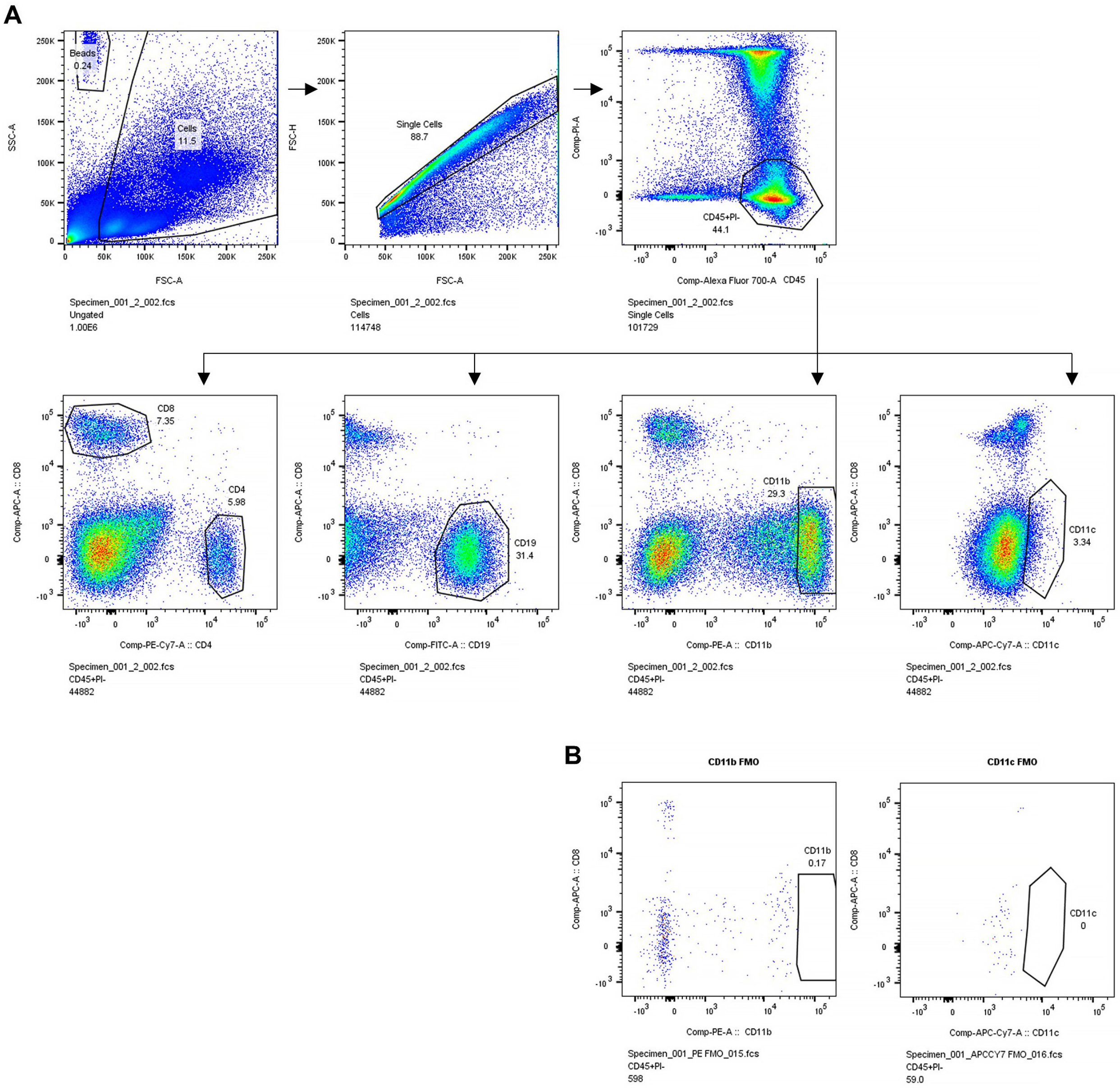

Supplementary Fig. 2: Gating strategy used to analyze circulating leukocyte composition following PLX5622 or vehicle. ***A,*** Gating strategy to determine proportions of blood leukocytes ($CD45^{+}PI^{-}$) expressing common lymphocyte (CD4, CD8, CD19) or myeloid (CD11b, CD11c) markers from 7-month-treated or Wild-type mice presented in Fig. 3A. ***B,*** Fluorescence minus one (FMO) controls showing background signal in PE (CD11b) and APC-Cy7 (CD11c) channels that were excluded from gating strategy in **(A)**.

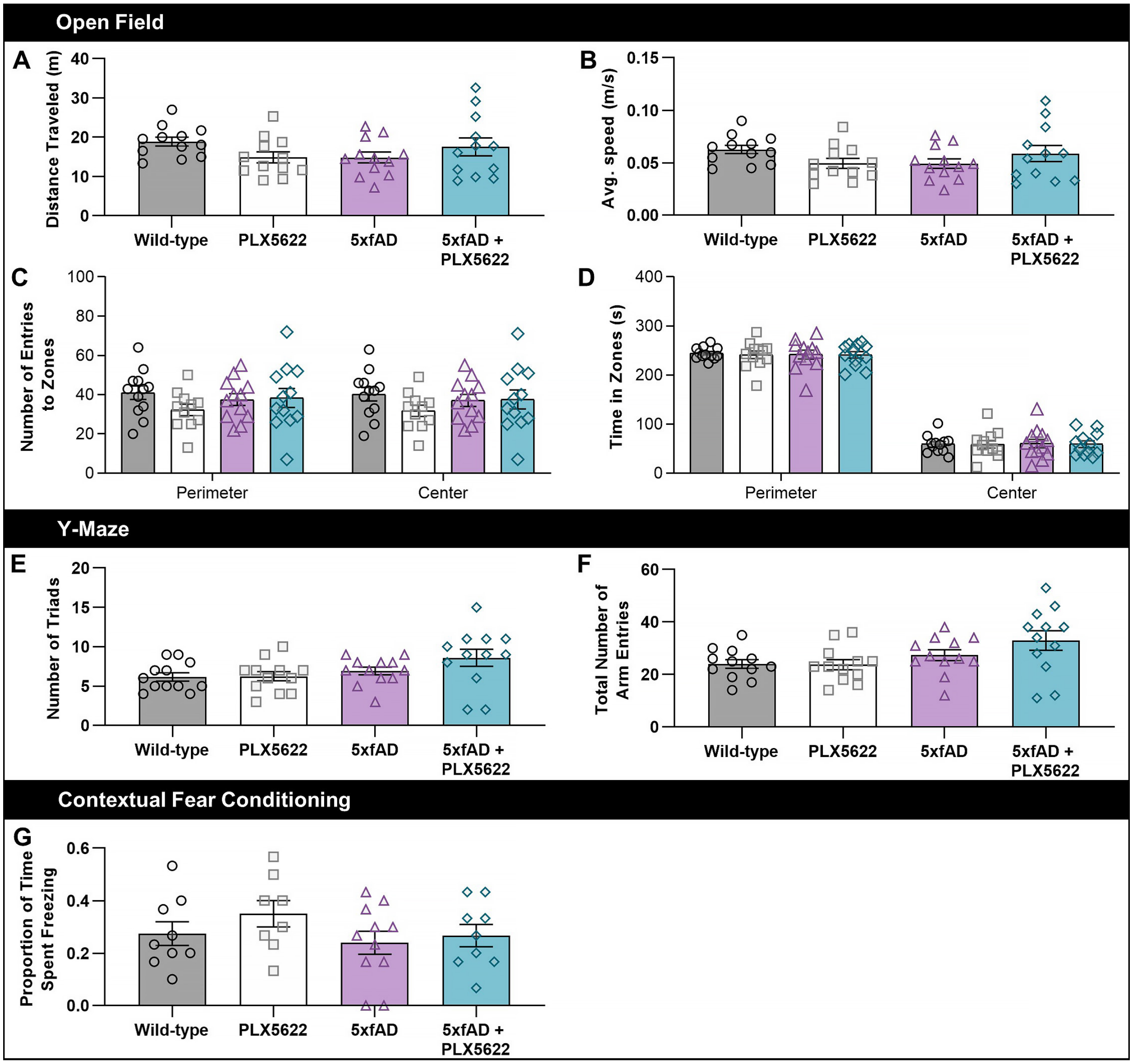

Supplementary Fig. 3: Effects of adult extended elimination of microglia on behavior and cognition. 7-month-old animals from all groups underwent behavioral assessment with Open Field (OF), Y-Maze, and Contextual fear conditioning (CFC). ***A-D,*** In OF, distance traveled (**A**), average speed (**B**), number of zone entries (**C**), and time spent in each zone (**D**) was unchanged between groups. ***E, F,*** In the Y-Maze, animals also exhibited unaltered performance, quantified as number of triads (**E**) and number of arm entries (**F**). ***G,*** No differences were detected between groups in CFC. Two-way ANOVA with Tukey's post hoc test for behavioral analyses. n=10-12 for Wild-

type, n=10-12 for PLX5622, n=11-12 for 5xFAD, n=9-12 for 5xFAD+PLX5622. Error bars indicate SEM.

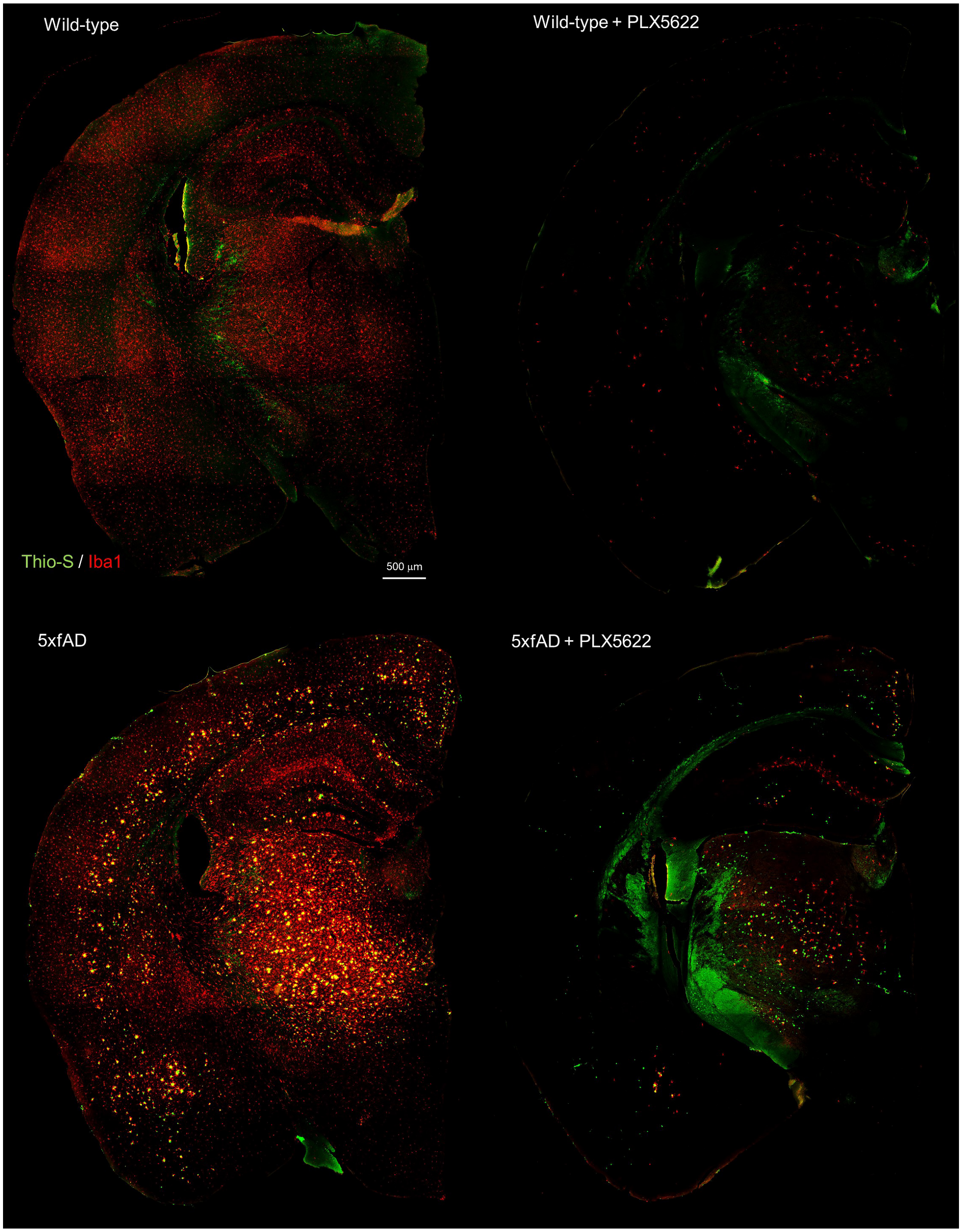

Supplementary Fig. 4: **The absence of microglia in 5xFAD mice prevents plaque formation.** Representative hemisphere stitches of dense-core deposits stained with Thioflavin-S (Thio-S) in green and microglia (IBA1 in red) show the formation of plaques solely in regions with surviving microglia. Scale bar: 500 µm.

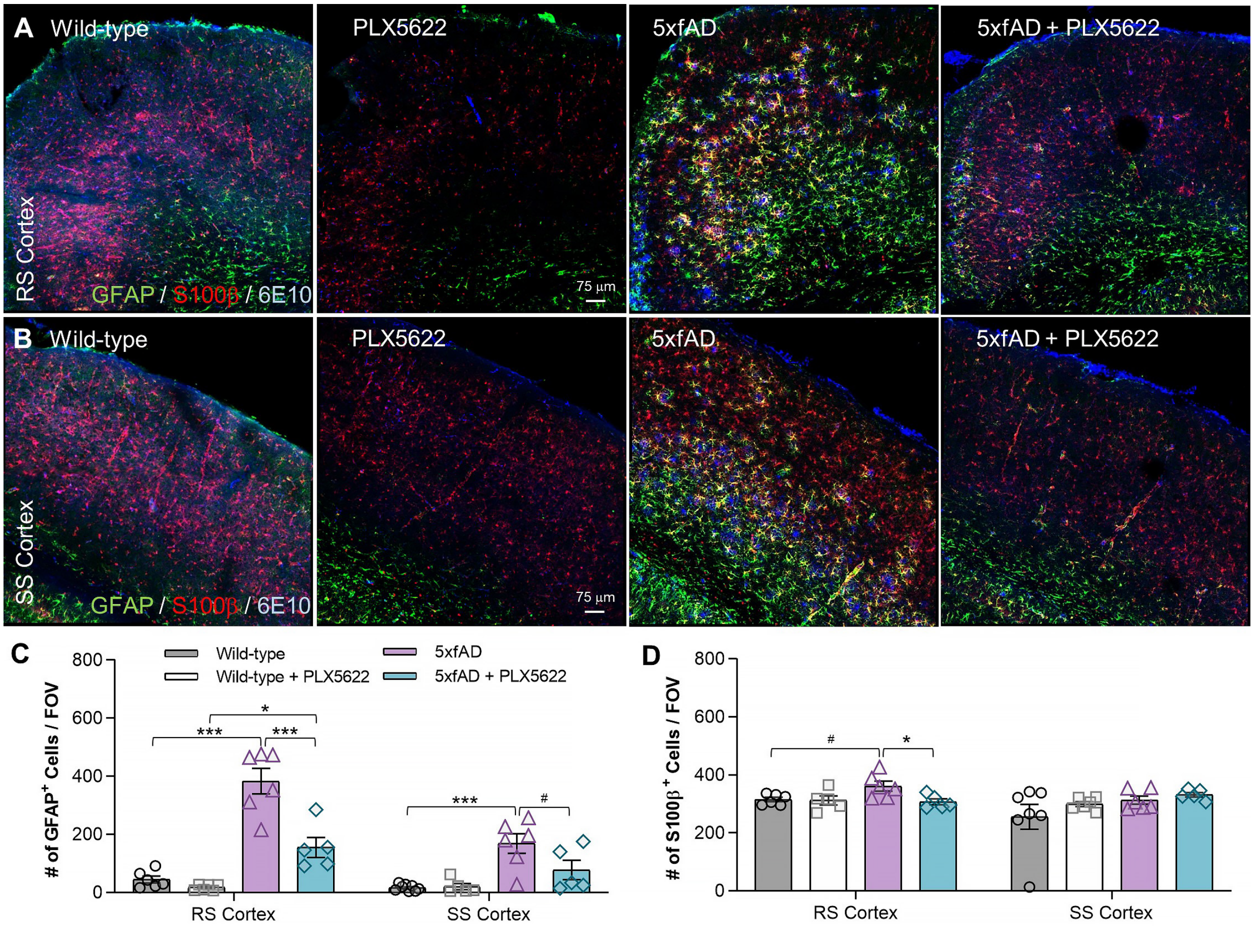

Supplementary Fig. 5: Reduced astrocytic responses in the absence of microglia. All analyses listed in respective order for retrosplenial (RS) cortex and somatosensory (SS) cortex. ***A, B,*** Confocal images of brain sections from each group immunolabeled for reactive astrocytes (GFAP in green and S100β in red) and diffuse plaques (6E10 in blue). ***C,*** Quantification of GFAP^{+} cell number (WT v 5xFAD, p<0.001, p<0.001; PLX5622 v 5xFAD+PLX5622, p=0.015, NS; 5xFAD v 5xFAD+PLX5622, p<0.001. p=0.055). Two-way ANOVA with Tukey's post hoc test for analyses. n=6 for Wild-type, n=6 for PLX5622, n=6 for 5xFAD, n=5 for 5xFAD+PLX5622. ***D,*** In the RS cortex, the number of S100β $^{+}$ astrocytes trended to an increase in 5xFAD animals compared to WT (p=0.081), with microglial elimination normalizing S100β number (p=0.044). Two-way ANOVA with Tukey's post hoc test for analyses. n=6 for Wild-type, n=5 for PLX5622,

n=6 for 5xFAD, n=5 for 5xFAD+PLX5622. Statistical significance is denoted by *$p<0.05$, **$p<0.01$, and ***$p<0.001$. Statistical trends are given as $^{\#}p<0.100$. Error bars indicate SEM. Scale bars: (A,B) 75 μm.

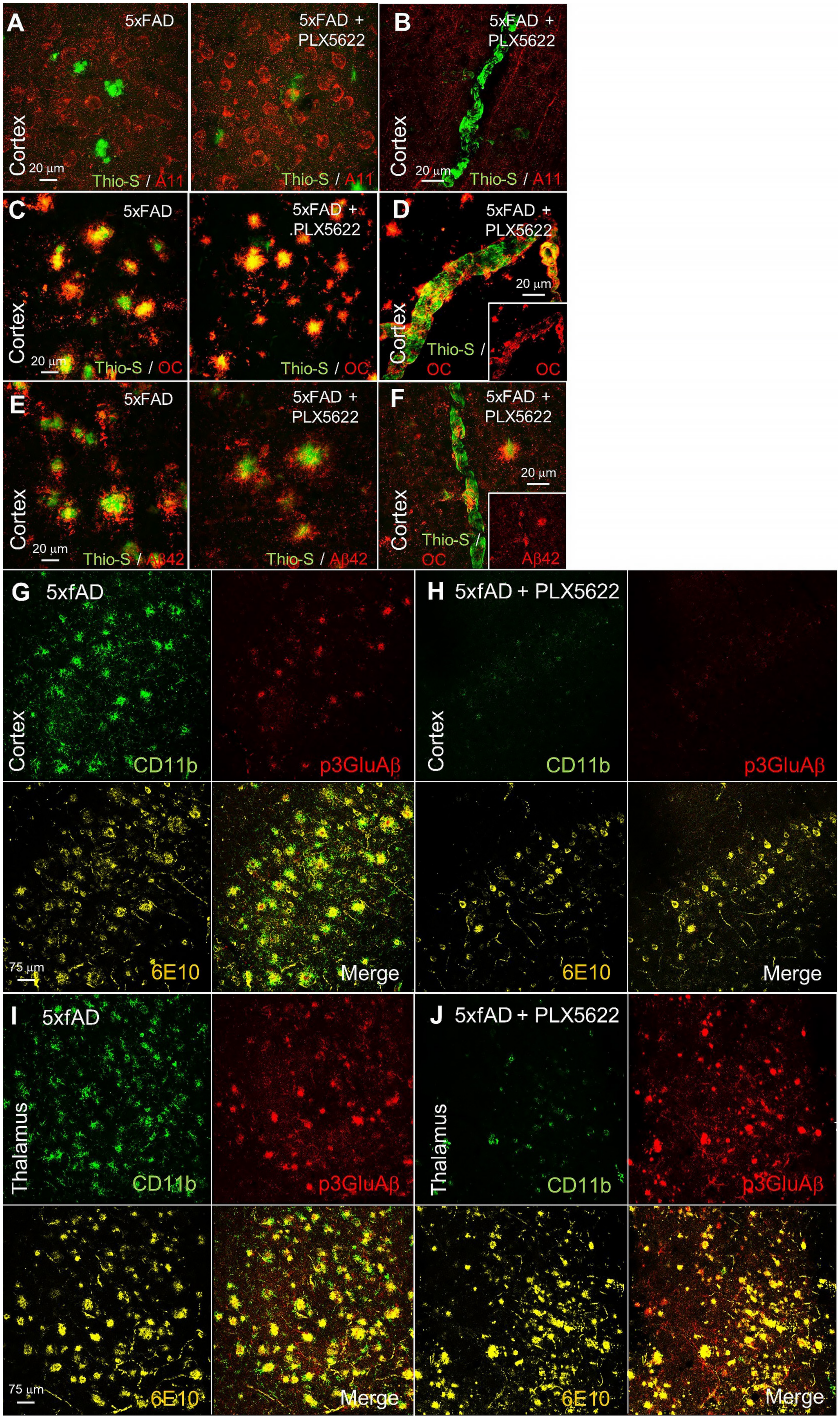

A
5xFAD
5xFAD + PLX5622
B
5xFAD + PLX5622
Cortex
20 µm
Thio-S / A11
C
5xFAD
5xFAD + PLX5622
D
5xFAD + PLX5622
Thio-S / OC
OC
E
5xFAD
5xFAD + PLX5622
F
5xFAD + PLX5622
Thio-S / Aβ42
Aβ42
G 5xfAD
H 5xfAD + PLX5622
CD11b
p3GluAβ
6E10
Merge
75 µm
I 5xfAD
J 5xfAD + PLX5622
Thalamus

Supplementary Fig. 6: Characterization of amyloid aggregates in the 5xFAD brain devoid of microglia. ***A, C, E,*** Immunolabeling for oligomers (A11), protofibrils (OC), and $A\beta_{1-42}$ revealed a high association of OC and $A\beta_{1-42}$ with parenchymal deposits (Thio-S in green), but not A11. ***B, D, F,*** Cortical sections of PLX5622-treated 5xFAD mice with vascular amyloid pathology, showing OC and $A\beta_{1-42}$ immunoreactive blood vessels. ***G-J,*** Confocal images of the cortex (**G-H**) and thalamus (**I-J**) of 5xFAD groups immunolabeled for microglia (CD11b in green), pyroglutamate-3 Aβ (p3GluAβ in red) and plaques (6E10 in yellow). Scale bars: (A,C,E) 20 μm; (B,D,F) 20 μm; (G-J) 75 μm.

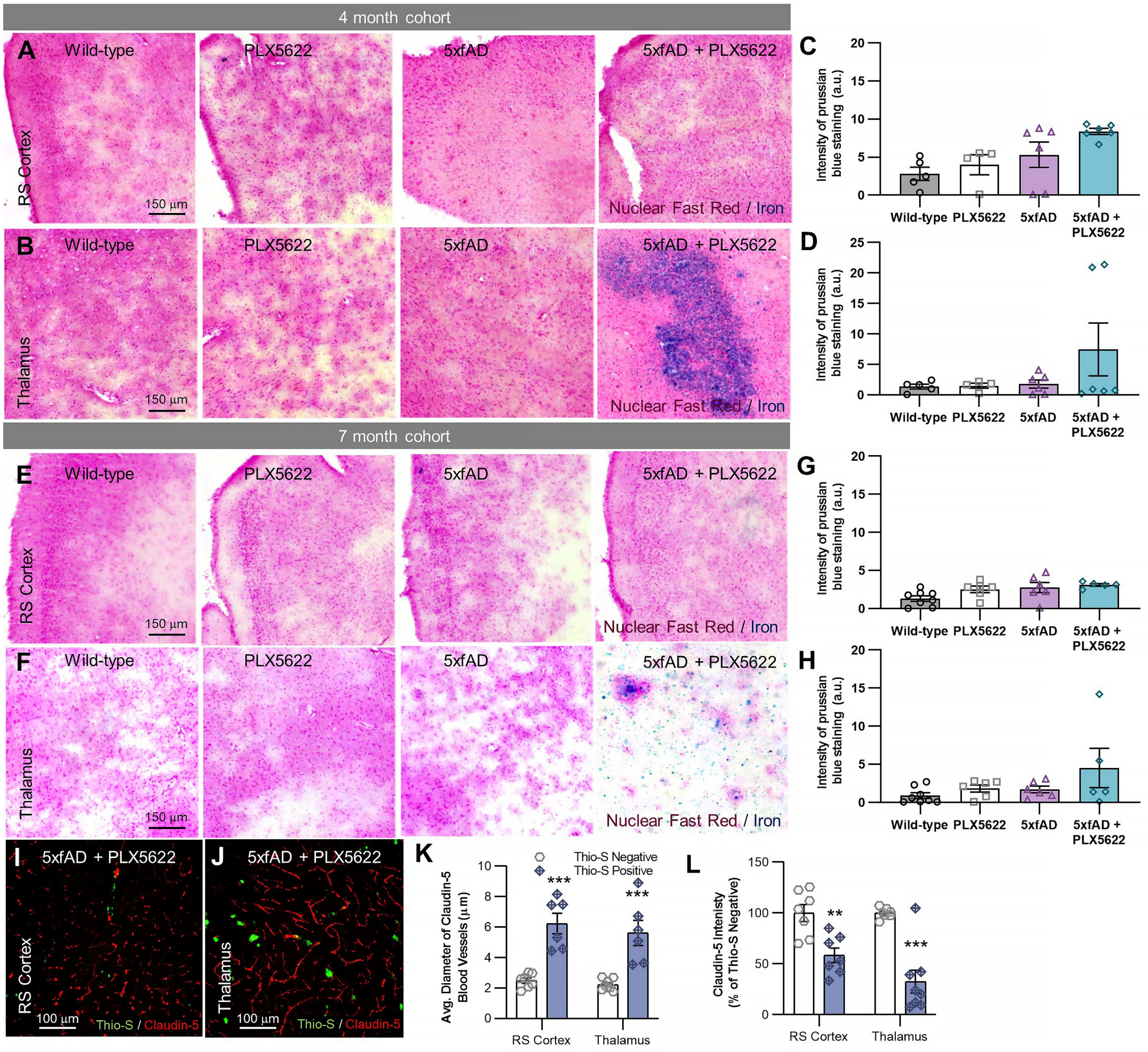

Supplementary Fig. 7: Evaluation of vascular Aβ accumulation on blood brain barrier disruption. All analyses listed in respective order for retrosplenial (RS) cortex and thalamus. ***A-H*,** Sections from 4- (**A-D**) and 7- (**E-H**) month old cohorts of mice underwent prussian blue staining of hemosiderin to evaluate microbleeds**.** Quantification of hemosiderin (*i.e.*, iron reactivity) in 4-(**C-D**) and 7-(**G-H**) month old animals revealed no differences between groups. Two-tailed independent t-test; n=5-8 for Wild-type; n=4-6 for PLX5622; n=6 for 5xFAD; n=5-6 for 5xFAD+PLX5622. ***I-J*,** Immunolabeling of 4-month-old 5xFAD animals treated with PLX5622 for

the endothelial marker, Claudin-5 (in red), and stained for aggregated Aβ (Thio-S in green). ***K,*** Claudin-5 diameter is increased in blood vessels associated with Aβ, relative to Thio-S negative blood vessels ($p<0.001$; $p<0.001$). Two-tailed independent t-test; n=6 for Thio-S^{+} blood vessels; n=7-8 for Thio-S^{-} blood vessels. ***L,*** Claudin-5^{+} blood vessel intensity is reduced when associated with aggregated Aβ ($p=0.003$; $p<0.001$). Two-tailed independent t-test; n=7 for Thio-S positive blood vessels; n=7-8 for Thio-S negative blood vessels. Statistical significance is denoted by *$p<0.05$, **$p<0.01$, and ***$p<0.001$. Error bars indicate SEM. Scale bars: (A,B) 150 μm; (E,F) 150 μm; (I,J) 100 μm.

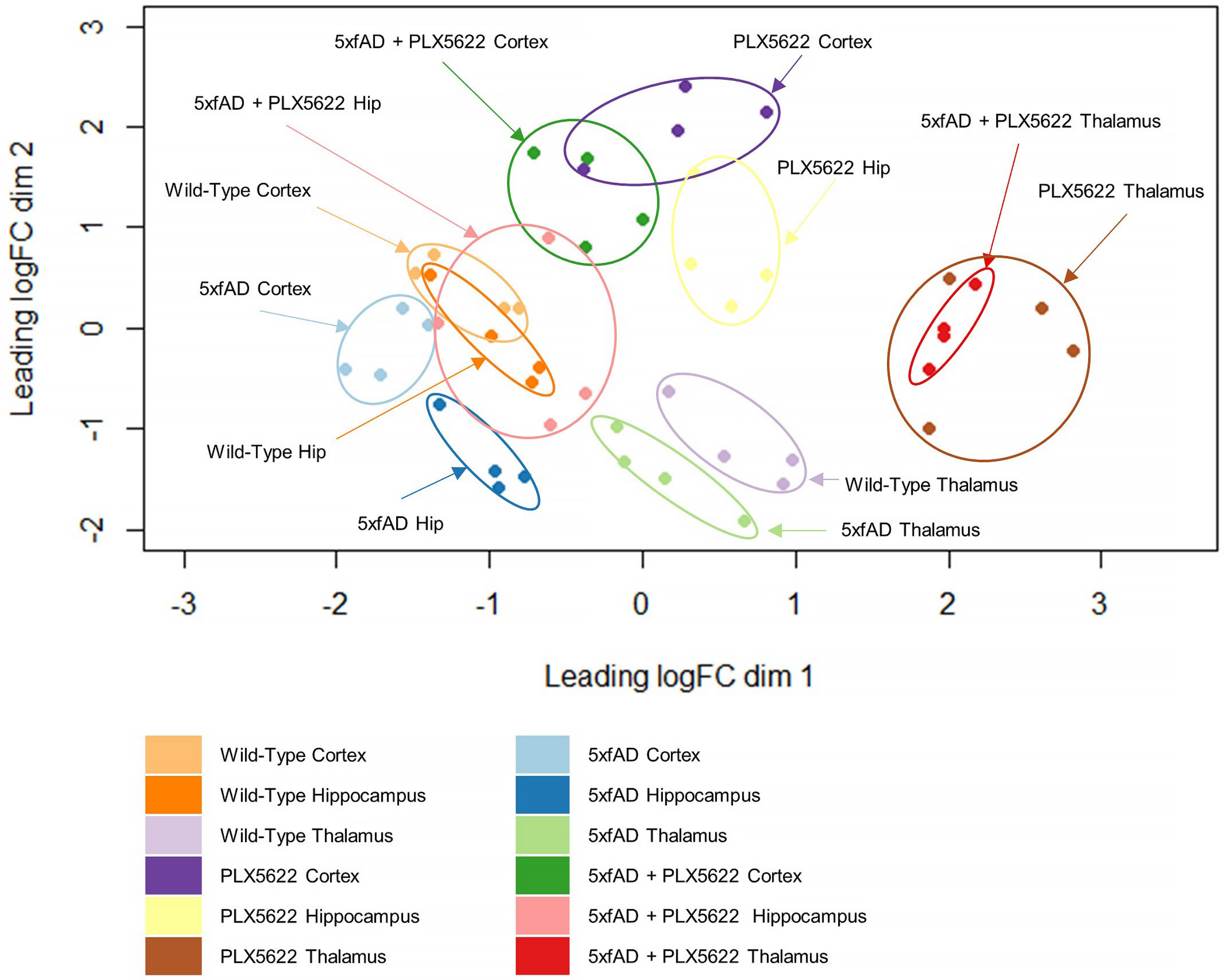

Supplementary Fig. 8: Principal component analysis of RNA-seq data. Principal component analysis of log counts per million mapped reads for each of the 12 groups (Wild-type Cortex, Hippocampus, and thalamus; PLX5622 Cortex, Hippocampus, and thalamus; 5xFAD Cortex, Hippocampus, and thalamus; and 5xFAD+PLX5622 Cortex, Hippocampus, and thalamus), after filtering genes that were not expressed and normalizing gene expression distributions. Groups denoted by color and arrows. n=4 for all groups.

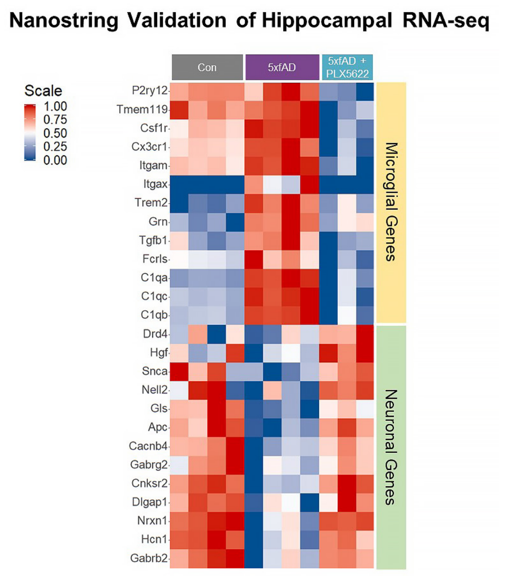

Supplementary Fig. 9: NanoString validation of RNA-seq data. Heatmap showing relative expression of genes associated with microglia or neurons, from hippocampal RNA extracted from Wild-type, PLX5622, 5xFAD, and 5xFAD+PLX5622 treated mice. n=4 for all groups.

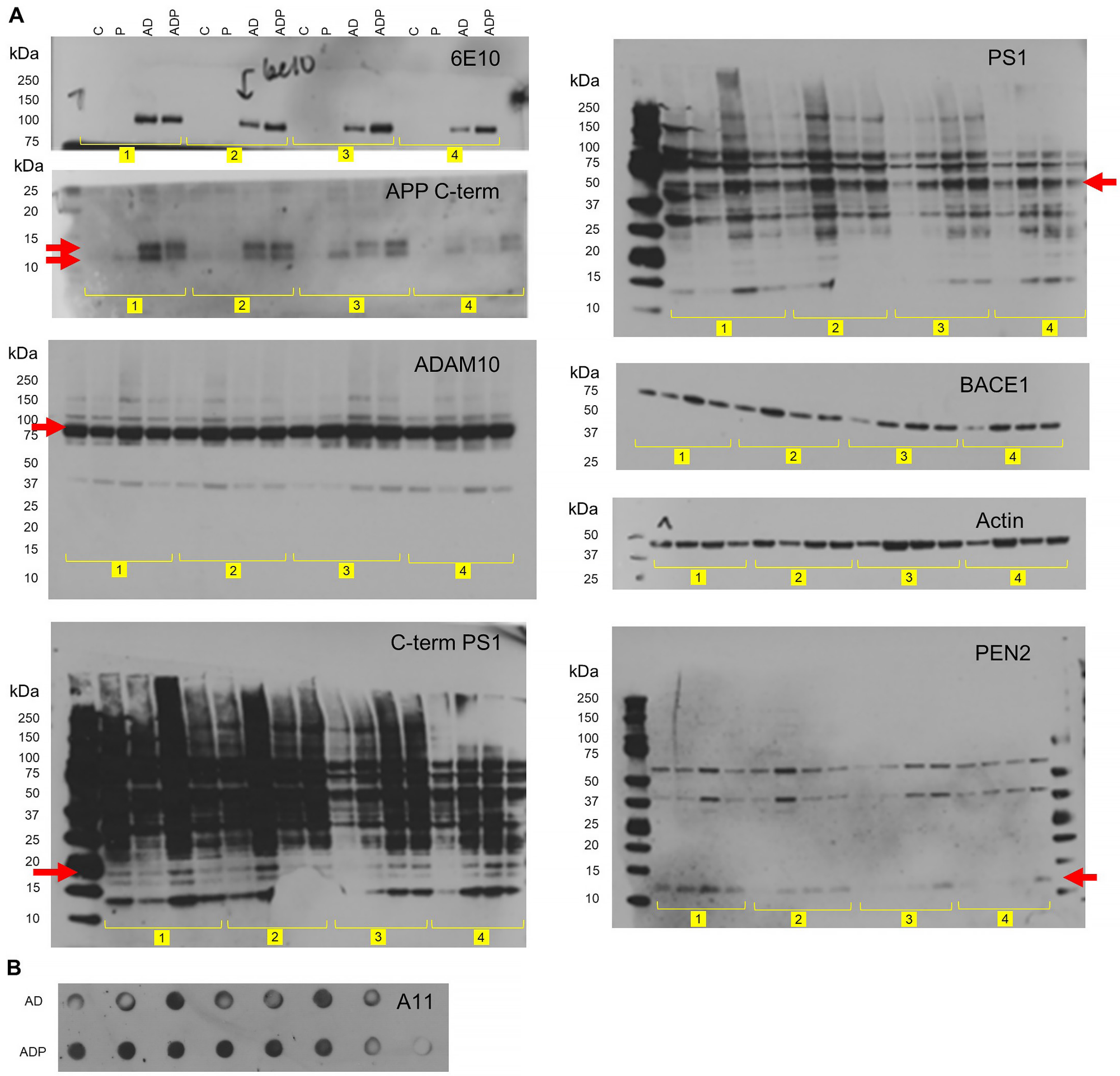

Supplementary Fig. 10: Uncropped scans of blots. ***A,*** Numbers in yellow box correspond to repetition number. When multiple bands are present on a western blot, red arrow indicates band of interest. Sequence of samples in each repetition is as follows: Control (C), PLX5622 (P), 5xFAD (AD), 5xFAD+PLX5622 (ADP). ***B,*** Uncropped image of A11 dot blot. Samples in each row are as follows: 5xFAD (AD), 5xFAD+PLX5622 (ADP).
