# Supplementary material for: Sustained microglial depletion with CSF1R inhibitor impairs parenchymal plaque development in an Alzheimer’s disease model
Source: Nat Commun. 2019 Aug 21;10:3758. doi: 10.1038/s41467-019-11674-z (PMC6704256; doi:10.1038/s41467-019-11674-z)
Supplement: Supplementary file 3 — Reporting Summary [file 41467_2019_11674_MOESM3_ESM.pdf]

nature research

Corresponding author(s): Kim Green

Reporting Summary

Nature Research wishes to improve the reproducibility of the work that we publish. This form provides structure for consistency and transparency in reporting. For further information on Nature Research policies, see Authors & Referees and the Editorial Policy Checklist.

Statistical parameters

When statistical analyses are reported, confirm that the following items are present in the relevant location (e.g. figure legend, table legend, main text, or Methods section).

n/a	Confirmed	
☐	☒	The exact sample size (n) for each experimental group/condition, given as a discrete number and unit of measurement
☐	☒	An indication of whether measurements were taken from distinct samples or whether the same sample was measured repeatedly
☐	☒	The statistical test(s) used AND whether they are one- or two-sided<br>*Only common tests should be described solely by name; describe more complex techniques in the Methods section.*
☐	☒	A description of all covariates tested
☐	☒	A description of any assumptions or corrections, such as tests of normality and adjustment for multiple comparisons
☐	☒	A full description of the statistics including central tendency (e.g. means) or other basic estimates (e.g. regression coefficient) AND variation (e.g. standard deviation) or associated estimates of uncertainty (e.g. confidence intervals)
☐	☒	For null hypothesis testing, the test statistic (e.g. F, t, r) with confidence intervals, effect sizes, degrees of freedom and P value noted<br>*Give P values as exact values whenever suitable.*
☒	☐	For Bayesian analysis, information on the choice of priors and Markov chain Monte Carlo settings
☒	☐	For hierarchical and complex designs, identification of the appropriate level for tests and full reporting of outcomes
☐	☒	Estimates of effect sizes (e.g. Cohen's d, Pearson's r), indicating how they were calculated
☒	☐	Clearly defined error bars<br>*State explicitly what error bars represent (e.g. SD, SE, CI)*

Our web collection on statistics for biologists may be useful.

Software and code

Policy information about availability of computer code

Data collection	Mouse behavior: ANY-maze 4.99<br>RNA-seq: HiSeq4000<br>Image acquisition: LASX v 3.3.0<br>Flow Cytometry: BD FACS AriaII<br>ELISA: Meso Scale Discovery Workbench v4.0
Data analysis	RNA-seq: STAR20 aligner, Rsubread21 package in R, edgeR23 package, limma24 package in R<br>Imaging analysis: Imaris v 9.2 and ImageJ<br>Flow cytometry analysis: FlowJo<br>Statistical analyses: Graphpad Prism Version 6, Microsoft Excel version 16.15, limma package in R

For manuscripts utilizing custom algorithms or software that are central to the research but not yet described in published literature, software must be made available to editors/reviewers upon request. We strongly encourage code deposition in a community repository (e.g. GitHub). See the Nature Research guidelines for submitting code & software for further information.

Data

Policy information about availability of data

All manuscripts must include a data availability statement. This statement should provide the following information, where applicable:

- Accession codes, unique identifiers, or web links for publicly available datasets
- A list of figures that have associated raw data
- A description of any restrictions on data availability

A searchable database with all RPKM values can be found at http://rnaseq.mind.uci.edu/green/ad_plx/gene_search.php. The atomic coordinates and structure factors of CSF1R in complex with PLX5622 (PDB accession code: 6N33) can be downloaded from the website of Protein Data Bank, www.rcsb.org. Additional data that support the findings of this study are available from the corresponding author upon reasonable request.

Field-specific reporting

Please select the best fit for your research. If you are not sure, read the appropriate sections before making your selection.

☒ Life sciences ☐ Behavioural & social sciences ☐ Ecological, evolutionary & environmental sciences

For a reference copy of the document with all sections, see nature.com/authors/policies/ReportingSummary-flat.pdf

Life sciences study design

All studies must disclose on these points even when the disclosure is negative.

Sample size	No statistical methods were used to predetermine sample sizes. However, our sample sizes are similar to those reported in previously published papers, in which these sample sizes have been demonstrated to be appropriate to detect significant effects. This is reported in the Statistics section, within the methods
Data exclusions	No data points were excluded. All sample sizes are reported throughout the text.
Replication	For each experimental condition, 3-4 regions of the brain were analyzed per animal and at least 3 animals were analyzed per condition. All attempts at replication were reproducible. For representative images, quantification of replicates is provided.
Randomization	Mice were randomized from each group of CSF1R-inhibitor-treated and control-treated animals. For all molecular, imaging, and behavioral experiments, animals were randomly assigned to groups.
Blinding	Blinding was performed for behavioral testing/analyses and during imaging. For all other analyses, investigators were not blinded to the conditions of the experiment.

Reporting for specific materials, systems and methods

Materials & experimental systems

n/a	Involved in the study	
☐	☒	Unique biological materials
☐	☒	Antibodies
☒	☐	Eukaryotic cell lines
☒	☐	Palaeontology
☐	☒	Animals and other organisms
☐	☒	Human research participants

Methods

n/a	Involved in the study	
☒	☐	ChIP-seq
☐	☒	Flow cytometry
☒	☐	MRI-based neuroimaging

Unique biological materials

Policy information about availability of materials

Obtaining unique materials	The CSF1R inhibitors used in this study, as well as control diet, were provided by Plexxikon.

Antibodies

Antibodies used

Flow Cytometry: anti-mouse CD16/32 (14-0161-81; eBioscience), A700 anti-mouse CD45 (103127; BioLegend), PE anti-mouse CD11b (101208; BioLegend), APC-Cy7 anti-mouse CD11c (117323; BioLegend), PE-Cy7 anti-mouse CD4 (100421; BioLegend), APC anti-mouse CD8 (100711; BioLegend) and FITC anti-mouse CD19 (115505; BioLegend).

Confocal Microscopy: anti-ionized calcium-binding adapter molecule 1 (IBA1; 1:1000; 019-19741; Wako, Osaka, Japan), anti-Aβ1-16 (6E10; 1:1000; 803001; BioLegend, San Diego, CA), anti-A11 oligomers (A11; 1:100; AHB0052; Thermo-Fisher Scientific), anti-amyloid fibrils OC (OC; 1:100; AB2286; EMD Millipore; Burlington, MA), anti-Aβ1-42 (1:200; ab10148; Abcam), anti-CD68 (1:500; MCA1957; Bio-Rad, Hercules, CA), anti-CD11b (1:50, MCA711; Bio-Rad), anti-CD31 (1:200; 550274; BD Pharmingen, San Diego, CA), anti-lysosomal associated membrane protein 1 (LAMP1; 1:200; sc-20011; Santa Cruz Biotechnology; Dallas, TX), anti-amyloid precursor protein, c-terminal (APP; 1:500; 171610; Calbiochem, San Diego, CA), anti-s100β (1:200; ab52642; Abcam, Cambridge, MA), anti-glial fibrillary protein (GFAP; 1:1000; ab4674; Abcam), anti-β-amyloid [pyroglutamate-3] (p3GluAβ; 1:500; NBP1-44048; Novus Biologicals; Littleton, CO), anti-apolipoprotein E (ApoE; 1:100; ab1906; Abcam), and anti-claudin-5 (1:500; 35-2500; Invitrogen).
Secondary antibodies conjugated to Alexa Fluor 488, 555, and 635 (Molecular Probes) were 1:500 diluted, including AF488 goat anti-rabbit (Invitrogen, A11029), AF555 goat anti-rabbit (Invitrogen, A21429), AF555 goat anti-mouse (Invitrogen, A21422), AF635 goat anti-mouse (Life Technologies, A31575), and AF635 goat anti-chicken (Life Technologies, A21103).

Immunoblotting: 6E10 (1:1000; 803001; BioLegend), anti-APP C-Terminal (APP; 1:1000; 171610; Calbiochem, San Diego, CA) for C99 and C83, anti-ADAM10 (1:1000; ab1997; Abcam), anti-β-secretase 1 (BACE1; 1:1000; ab2077; Abcam), anti-Presenilin-1 (PS1; 1:1000; ab76083; Abcam), anti-Presenilin enhancer 2 (PEN2; 1:500; ab18189; Abcam), and anti-β-actin (1:10,000; MA5-15739; Sigma-Aldrich).

Validation

The antibodies used for flow cytometry are widely used and were validated in the listed studies.
The CD45 antibody is widely used and was validated in:
Feng Tian, Wenlong Yang, Daniel A. Mordes, Jin-Yuan Wang, Johnny S. Salameh, Joanie Mok, Jeannie Chew, Aarti Sharma, Ester Leno-Duran, Satomi Suzuki-Uematsu, Naoki Suzuki, Steve S. Han, Fa-Ke Lu, Minbiao Ji, Rosanna Zhang, Yue Liu, Jack Strominger, Neil A. Shneider, Leonard Petrucelli, X. Sunney Xie, Kevin Eggan (2016). Monitoring peripheral nerve degeneration in ALS by label-free stimulated Raman scattering imaging. Nat Commun. 7: 13283. doi: 10.1038/ncomms13283

The CD11c antibody is widely used and was validated in:
Shahzada Khan, Erik M. Woodruff, Martin Trapecar, Krystal A. Fontaine, Ashley Ezaki, Timothy C. Borbet, Melanie Ott, Shomyseh Sanjabi (2016). Dampened antiviral immunity to intravaginal exposure to RNA viral pathogens allows enhanced viral replication. J Exp Med. 213(13): 2913–2929. doi: 10.1084/jem.20161289

The CD8 antibody is widely used and was validated in:
Thomas Riffelmacher, Alexander Clarke, Felix C. Richter, Amanda Stranks, Sumeet Pandey, Sara Danielli, Philip Hublitz, Zhanru Yu, Errin Johnson, Tobias Schwerd, James McCullagh, Holm Uhlig, Sten Eirik W. Jacobsen, Anna Katharina Simon (2017). Autophagy-Dependent Generation of Free Fatty Acids Is Critical for Normal Neutrophil Differentiation. Immunity. 47(3): 466–480.e5. doi: 10.1016/j.immuni.2017.08.005

The CD4 antibody is widely used and was validated in:
Harald Prüss, Andrea Tedeschi, Aude Thiriot, Lydia Lynch, Scott M. Loughhead, Susanne Stutte, Irina B. Mazo, Marcel A. Kopp, Benedikt Brommer, Christian Blex, et al. (2017). Spinal cord injury-induced immunodeficiency is mediated by a sympathetic-neuroendocrine adrenal reflex. Nat Neurosci. doi: 10.1038/nn.4643

The CD16/32 antibody is widely used and was validated in:
Aurélie Hérault, Mikhail Binnewies, Stephanie Leong, Fernando J. Calero-Nieto, Si Yi Zhang, Yoon-A Kang, Xiaonan Wang, Eric M. Pietras, S. Haihua Chu, Keegan Barry-Holson, Scott Armstrong, Berthold Göttgens, Emmanuelle Passegué (2017). Myeloid progenitor cluster formation drives emergency and leukemic myelopoiesis. Nature. 544(7648): 53–58. Published online 2017 Mar 29. doi: 10.1038/nature21693

The CD11b antibody is widely used and was validated in:
Thomas Riffelmacher, Alexander Clarke, Felix C. Richter, Amanda Stranks, Sumeet Pandey, Sara Danielli, Philip Hublitz, Zhanru Yu, Errin Johnson, Tobias Schwerd, James McCullagh, Holm Uhlig, Sten Eirik W. Jacobsen, Anna Katharina Simon (2017). Autophagy-Dependent Generation of Free Fatty Acids Is Critical for Normal Neutrophil Differentiation. Immunity. 47(3): 466–480.e5. doi: 10.1016/j.immuni.2017.08.005

The CD19 antibody is widely used and was validated in:
Eden Kleiman, Haiqun Jia, Salvatore Loguercio, Andrew I. Su, Ann J. Feeney (2016). YY1 plays an essential role at all stages of B-cell differentiation. Proc Natl Acad Sci U S A. 113(27): E3911–E3920. doi: 10.1073/pnas.1606297113

For immunohistochemistry and biochemistry, IBA1, 6E10, CD68, S100β, GFAP, APP C-Terminal, β-actin, along with the listed secondary antibodies, are widely used and were validated in our previous studies:
Rachel A. Rice, Jason Pham, Rafael J. Lee, Allison R. Najafi, Brian L. West, Kim N. Green (2017). Microglial repopulation resolves inflammation and promotes brain recovery after injury. Glia. 65(6): 931–944. doi: 10.1002/glia.23135

Monica Renee Pittman Elmore, Allison Rachel Najafi, Maya Allegra Koike, Nabil Nazih Dagher, Elizabeth Erin Spangenberg, Rachel Anne Rice, Masashi Kitazawa, Bernice Matusow, Hoa Nguyen, Brian Lee West, Kim Nicholas Green (2014). CSF1 receptor signaling is necessary for microglia viability, which unmasks a cell that rapidly repopulates the microglia-depleted adult brain.

Neuron. 82(2): 380–397. doi: 10.1016/j.neuron.2014.02.040

Elizabeth E. Spangenberg, Rafael J. Lee, Allison R. Najafi, Rachel A. Rice, Monica R. P. Elmore, Mathew Blurton-Jones, Brian L. West, Kim N. Green (2016). Eliminating microglia in Alzheimer's mice prevents neuronal loss without modulating amyloid-β pathology. Brain. 139(4): 1265–1281. doi: 10.1093/brain/aww016

The OC antibody is widely used and was validated in:
Kayed, R., Head, E., Sarsoza, F., Saing, T., Cotman, C. W., Necula, M, Glabe, C. G. (2007). Fibril specific, conformation dependent antibodies recognize a generic epitope common to amyloid fibrils and fibrillar oligomers that is absent in prefibrillar oligomers. Molecular Neurodegeneration, 2, 18. http://doi.org/10.1186/1750-1326-2-18

The Aβ1-42 antibody is widely used and was validated in:
Khan, A. A., Mao, X. O., Banwait, S., Jin, K., & Greenberg, D. A. (2007). Neuroglobin attenuates β-amyloid neurotoxicity in vitro and transgenic Alzheimer phenotype in vivo. Proceedings of the National Academy of Sciences of the United States of America, 104(48), 19114–19119. http://doi.org/10.1073/pnas.0706167104

The Aβ [pyro glu3] antibody is widely used and was validated in:
W. He, C. J. Barrow (1999). The A beta 3-pyroglutamyl and 11-pyroglutamyl peptides found in senile plaque have greater beta-sheet forming and aggregation propensities in vitro than full-length A beta. Biochemistry. 38(33): 10871–10877. doi: 10.1021/bi990563r

The CD11b antibody is widely used and was validated in:
Muktha S. Natrajan, Alerie G. de la Fuente, Abbe H. Crawford, Eimear Linehan, Vanessa Nuñez, Kory R. Johnson, Tianxia Wu, Denise C. Fitzgerald, Mercedes Ricote, Bibiana Bielekova, Robin J. M. Franklin (2015). Retinoid X receptor activation reverses age-related deficiencies in myelin debris phagocytosis and remyelination. Brain. 138(12): 3581–3597. doi: 10.1093/brain/awv289

The CD31 antibody is widely used and was validated in:
Hagen Kunte, Markus A. Busch, Katrin Trostdorf, Bernd Vollnberg, Lutz Harms, Rupal Mehta, Rudolf J. Castellani, Pitchaiah Mandava, Thomas A. Kent, J. Marc Simard (2012). Hemorrhagic transformation of ischemic stroke in diabetics on sulfonylureas.Ann Neurol. 72(5): 799–806. doi: 10.1002/ana.23680

The LAMP1 antibody is widely used and was validated in:
Carlo Condello, Peng Yuan, Aaron Schain, Jaime Grutzendler (2015). Microglia constitute a barrier that prevents neurotoxic protofibrillar Aβ42 hotspots around plaques. Nat Commun. 6: 6176. doi: 10.1038/ncomms7176

The ApoE antibody is widely used and was validated in:
Nora Pencheva, Colin G. Buss, Jessica Posada, Taha Merghoub, Sohail F. Tavazoie (2014). Borad-spectrum therapeutic suppression of metastatic melanoma through nuclear hormone receptor activation. Cell. 156(5): 986-1001. doi: 10.1016/j.cell.2014.01.038

The Claudin-5 antibody is widely used and was validated in:
Keisuke Yanagida, Catherine H. Liu, Giuseppe Faraco, Sylviam Galvani, Helen K. Smith, Nathalie Burg, Josef Anrather, Teresa Sanchez, Costantino Iadecola, Timothy Hla (2017). Size-selective opening of the blood-brain barrier by targeting endothelial sphingosine 1-phosphate receptor 1. PNAS 114(17): 4531-4536.

The ADAM10 antibody is widely used and was validated in:
Michael Klingener, Manideep Chavali, Jagdeep Singh, Nadia McMillan, Alexandra Coomes, Peter J. Dempsey, Emily I. Chen, Adan Aguirre (2014). N-Cadherin Promotes Recruitment and Migration of Neural Progenitor Cells from the SVZ Neural Stem Cell Niche into Demyelinated Lesions.J Neurosci. 34(29): 9590–9606. doi: 10.1523/JNEUROSCI.3699-13.2014

The BACE1 antibody is widely used and was validated in:
Tao Ma, Mimi A. Trinh, Alyse J. Wexler, Clarisse Bourbon, Evelina Gatti, Philippe Pierre, Douglas R. Cavener, Eric Klann (2013). Suppression of eIF2α kinases alleviates AD-related synaptic plasticity and spatial memory deficits. Nat Neurosci. 16(9): 1299–1305. doi: 10.1038/nn.3486

The PS1 antibody is widely used and was validated in:
Gunjan Joshi, Youjian Chi, Zheping Huang, Yanzhuang Wang. Aβ-induced Golgi fragmentation in Alzheimer's disease enhances Aβ production (2014). Proc Natl Acad Sci U S A. 111(13): E1230–E1239. doi: 10.1073/pnas.1320192111

The PEN2 antibody is widely used and was validated in:
Gizem Donmez, Diana Wang, Dena E. Cohen, Leonard Guarente (2010). SIRT1 Suppresses β-amyloid Production by Activating the α-Secretase Gene ADAM10. Cell.142(2): 320–332. doi: 10.1016/j.cell.2010.06.020

Animals and other organisms

Policy information about studies involving animals; ARRIVE guidelines recommended for reporting animal research

Laboratory animals	3xTg-AD (MMRRC Stock No: 34830-JAX) and 5xfAD mice (MMRRC Stock No: 34848-JAX), along with non-transgenic controls were used in these studies. 3, 4, and 7 month old animals were used for 5xfAD studies and groups were sex balanced. For 3xTg-AD animals, 15 month old males were analyzed.
Wild animals	This study did not involve wild animals.

Field-collected samples | This study did not involve field-collected samples.

Human research participants

Policy information about studies involving human research participants

Population characteristics	Human post-mortem tissue from non-demented, non-demented high pathology, and AD subjects was obtained from the Alzheimer's Disease Research Center, UC Irvine, with consent from the UC Irvine Ethics Committee. Neuropathological examination included Braak and Braak staging for plaques and tangles and diagnosis of neuropathological AD using National Institute on Aging-Reagan criteria.
Recruitment	Human participants were recruited by the Alzheimer's Disease Research Center at UC Irvine.

Flow Cytometry

Plots

Confirm that:

☒ The axis labels state the marker and fluorochrome used (e.g. CD4-FITC).

☒ The axis scales are clearly visible. Include numbers along axes only for bottom left plot of group (a 'group' is an analysis of identical markers).

☒ All plots are contour plots with outliers or pseudocolor plots.

☒ A numerical value for number of cells or percentage (with statistics) is provided.

Methodology

Sample preparation	Blood was drawn from mice via cardiac puncture and incubated with ammonium-chloride-potassium lysing buffer for red blood cell lysis. Blood leukocytes were subsequently blocked with anti-mouse CD16/32 (14-0161-81; eBioscience) and stained with fluorophore-conjugated antibodies A700 anti-mouse CD45 (103127; BioLegend), PE anti-mouse CD11b (101208; BioLegend), APC-Cy7 anti-mouse CD11c (117323; BioLegend), PE-Cy7 anti-mouse CD4 (100421; BioLegend), APC anti-mouse CD8 (100711; BioLegend) and FITC anti-mouse CD19 (165505; BioLegend), as well as propidium iodide (PI; 00-6990-50; eBioscience) for live/dead discrimination.
Instrument	BD FACSAria II (BD Biosciences)
Software	Flow Jo v7.6.1
Cell population abundance	At least 250,000 events were captured by the cytometer and 30,000 single cells analyzed per group. Dead cells were excluded as propidium iodide (PI)-positive.
Gating strategy	Gating was performed following routinely used protocols, using FSC/SSC to exclude dead cells, cell debris and doublets and propidium iodide to identify dead cells. Populations of live singlets were then gated was percentages of CD45+PI- cells.

☒ Tick this box to confirm that a figure exemplifying the gating strategy is provided in the Supplementary Information.
