# Supplementary figures and images for: Sustained microglial depletion with CSF1R inhibitor impairs parenchymal plaque development in an Alzheimer’s disease model
Source: Nat Commun. 2019 Aug 21;10:3758. doi: 10.1038/s41467-019-11674-z (PMC6704256; doi:10.1038/s41467-019-11674-z)

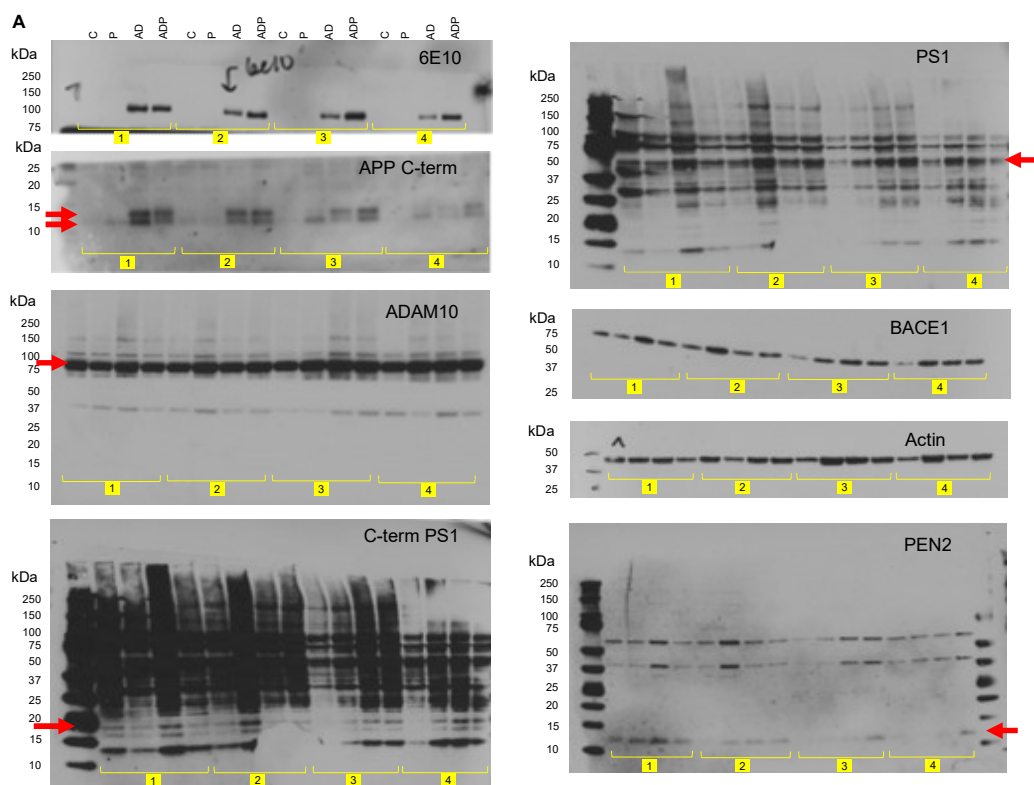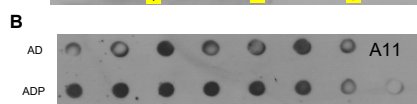

Supplement: Supplementary file 4 — Source Data [file 41467_2019_11674_MOESM4_ESM.zip › Source Data 1.pdf]
